# Measurement of Quality of Life VI. Quality-Adjusted Life Years (QALY) is an Unfortunate Use of the Quality-of-Life Concept

**DOI:** 10.1100/tsw.2003.79

**Published:** 2003-10-13

**Authors:** Soren Ventegodt, Joav Merrick, Niels Jorgen Andersen

**Affiliations:** The Quality of Life Research Center, Copenhagen K, Denmark

**Keywords:** Quality of Life, QOL, SEQOL, QOL5, QOL1, measurement, human development, holistic medicine, public health, Denmark

## Abstract

The QALY (quality-adjusted life years) attempts to incorporate the dimension of quality of life into the evaluation by adjusting life years by a quality factor. In practice, this is based on discussing with people the progression of a number of hypothetical illnesses and their ensuing side effects. From this information, the person assesses how each state of health described compares with a theoretical maximum state of health. For example, 1 day with a certain condition might the equivalent of living only 0.5 days in good health.We believe that QALY value only represents a superficial impression of a person's quality of life. In short, the QALY does not express what it means for a person to live a life at reduced quality. We believe that if the patients were optimally informed and allowed to decide for themselves, they would more often reject high-tech expensive biomedical treatments that only serve to prolong life and do not increase its quality. The problem of priorities may then turn out to be far more simple and also more ethical: the focus will be on the quality of life, not on QALY, and the question of the meaning of life and death will achieve greater openness and respect.

## INTRODUCTION

The primary goal of the medical profession is and always has been to keep the patient alive. As a result, the success of the physician has been judged in terms of how long a person lives after treatment (a purely quantitative measure). However, a consensus has been growing among health and social workers that a crucial dimension is missing in this evaluation: the quality of life (QOL). In an attempt to give the quality of life a more significant role in determining healthcare priorities, the concept of quality-adjusted life years (QALY) has been developed and used to varying degrees internationally for a number of years[1,2,3,4].

## QUALITY-ADJUSTED LIFE YEARS

QALY attempts to incorporate the dimension of quality of life into the evaluation by adjusting life years by a quality factor. In practice, this is based on discussing with people the progression of a number of hypothetical illnesses and their ensuing side effects. From this information, the person assesses how each state of health described compares with a theoretical maximum state of health. For example, 1 day with a certain condition might the equivalent of living only 0.5 days in good health.

Thus, 8 years with a quality of life considered to be only half as good as the theoretical maximum is expressed as (8 multiplied by 0.5 =) 4 quality-adjusted life years. These 8 life years correspond to 4 life years with maximum quality of life and 16 years with 25% quality of life. In reality, a person's quality of life tends to decline during his or her final years so that the QALY entity becomes the integral of this function or the area below the curve.

In the common situation in which not everyone can be offered the same treatment (for example, because of lack of funds, hearts for transplants, or medical staff) the physician, as the decision maker will, all things being equal, choose the treatment that results in the highest QALY score for the person. In turn, the doctor will select people for treatment whose QALY score after treatment is expected to be higher than that of other people. In other words, the hearts are given to those who are expected to benefit the most from them.

## IS QALY A GOOD MEASUREMENT?

Unlike other critics who have questioned the use at all of QALY[5], we believe that the problem of the QALY is not so much that it is used. The health sector always has to make choices, and the QALY score represents a more subtle reflection of life after treatment than the simpler measure of number of life years. We therefore feel that the QALY concept is a step in the right direction if the aim of treating the person is to enhance his or her quality of life. Nevertheless, we find that the QALY yardstick is too unsophisticated to use in setting priorities. It is not evidently reasonable to simply multiply time span by the quality of life.

Experienced time differs significantly from chronological time, and a person living a life with low quality can consider it a fate worse than death and there are some people who choose to commit suicide rather than live a life of low quality. Moreover, one fantastic year (1 year at 100% adjustment) can easily be more valuable than 50 years where one is barely alive, tottering on the brink of death with a quality of life of, say, 10% (50 years at 10% = 5 QALY).

Finally, there are still no quality-of-life rating scales that express the quality of life in the depths of the individual. So the QALY value only represents a superficial impression of a person's quality of life. In short, the QALY does not express what it means for a person to live a life at reduced quality.

## AN EXAMPLE

Another problem can be illustrated by a hypothetical example. Suppose that we investigate 1,000 people with breast cancer using our quality-of-life questionnaire SEQOL[5] and find that people receiving treatment A (for example, a combination of surgery, chemotherapy, and radiotherapy) had their quality of life reduced to 60% of what it was prior to treatment and that they continued to live in this state for 10 years (= 6 QALY). People who received treatment B (for example, only minor surgery) had their quality of life reduced to 80% of what it was prior to treatment, but only lived for 3 years (= 2.4 QALY). If the health sector uses such scores and calculations in making their priorities, they could argue that treatment A, rather than treatment B, ought to be chosen.

Nevertheless, this does not mean that Mrs. Jones should be given treatment A. What if Mrs. Jones considers her life to be pointless if the quality of life is less than 80%, the outcome of surgery? What if she objects to an aggressive treatment regimen for religious, ideological, or existential reasons? Or what if her hair and general appearance are all-important to her?

A thorough quality-of-life questionnaire like SEQOL and the concept of QALY can be used as an excellent starting point for a discussion of the problems, but is not a key to solving the problems. Life cannot be reduced to a mere number, and every situation in life is so complex that, ideally, every individual should make a personal decision on his or her medical treatment.

Even based on a global measure like SEQOL, QALY is still too limited an entity for taking fair medical decisions. No matter how well it is used, it can only reflect life at the surface, failing to unravel the deepest, and truest, sense of life and how each of us sees it.

## PRIORITIES WITH HARD CHOICES TO MAKE

The health sector needs to address the problems of rationing healthcare resources and setting priorities. Despite advances in technology and medicine, a lack of funds, both now and in the future, means that not everyone can enjoy the benefits of these advances. Physicians and politicians are already making daily choices to determine who gets what treatment and where and justify these decisions to the people they serve.

Politicians may choose to use the global and generic quality-of-life measures like SEQOL[5], or the more practical QOL5 and QOL1[6], to decide what treatment to give Mrs. Jones. By doing so they are presumably better off than not using any guideline at all for priority, but a much better alternative still is for the politician to engage in the demanding quest for truth and understanding of human life, which constitutes the very essence of the quality-of-life concept.

Instead of using numbers, choices are better based on understanding and wisdom. But let us face an unpleasant fact: in many cases the politicians are solving problems that the medical doctors should have solved themselves. Brave and wise doctors with compassionate hearts deeply rooted in the quality-of-life philosophy and minds focused by the intent of doing the best for all, and loaded with all the necessary knowledge, can and must take the necessary, often unpleasant, decisions of priority. QALY numbers *can* be a valuable guideline if the quality-of-life measure is well chosen for the purpose, but it can never substitute for wisdom.

## SOME THOUGHTS

Interestingly, focusing on the inner depths of the being means that every individual and his or her family are capable of make decisions on treatment also. These decisions are not only about what kind of treatment the patient needs (i.e., biomedical or alternative), but also about receiving the treatment at all. We believe that if the patients were optimally informed and were allowed to decide for themselves, they would more often reject high-tech expensive biomedical treatments that only serve to prolong life and do not increase its quality.

The problem of priorities may then turn out to be far more simple and also more ethical: the focus will be on the quality of life, not on QALY, and the question of the meaning of life and death will achieve greater openness and respect.

## BIOSKETCHES

**Søren Ventegodt, MD,** is the Director of the Quality of Life Research Center in Copenhagen, Denmark. He is also responsible for a Research Clinic for Holistic Medicine in Copenhagen and is a popular speaker throughout Scandinavia. He has published numerous scientific or popular articles and a number of books on holistic medicine, quality of life, and quality of working life. His most important scientific contributions are the comprehensive SEQOL questionnaire, the very short QOL5 questionnaire, the integrated quality-of-life (IQOL) theory, the holistic process theory, the life mission theory, and the Danish Quality of Life Research Survey, 1991–94 in cooperation with the University Hospital of Copenhagen and the late pediatric professor Bengt Zachau-Christiansen. E-mail: ventegodt@livskvalitet.org. Website: www.livskvalitet.org/

**Joav Merrick, MD, DMSc,** is Professor of Child Health and Human Development affiliated with the Zusman Child Development Center and Division of Community Health at the Ben Gurion University, Beer-Sheva, Israel and presently the Medical Director of the Division for Mental Retardation, Ministry of Social Affairs, Jerusalem and the Director of the National Institute of Child Health and Human Development. He has numerous publications in the field of child and human development, rehabilitation, intellectual disability, disability, health, welfare, abuse, advocacy and prevention. Dr. Merrick received the Peter Sabroe Child Award for outstanding work on behalf of Danish Children in 1985 and the International LEGO-Prize ("The Children's Nobel Prize") for an extraordinary contribution towards improvement in child welfare and well being in 1987. E-mail: jmerrick@internet-zahav.net. Website: www.nichd-israel.com

**Niels Jørgen Andersen, MSc,** Professor, Department of Innovation and Economic Organization, Norwegian School of Management. This department conducts research and provides teaching in central topics related to innovation, business development, management of global companies, business history, and economic organization. Research activities within the Department are related to four core subjects within the discipline: business history, cooperative organizations, business development and entrepreneurship, and finally studies of industries with a special focus on the electricity industry. He is also the dynamic chairman of the nonprofit organization Stiftelsen Holistisk Medisin Scandinavia, which aims to support the scientific development, research, and documentation of complementary and holistic medicine in Scandinavia. E-mail: niels.j.andersen@bi.no. Website: www.bi.no/users/fgl93013/
